# Animal models for investigating chronic pancreatitis

**DOI:** 10.1186/1755-1536-4-26

**Published:** 2011-12-01

**Authors:** Alexander A Aghdassi, Julia Mayerle, Sandra Christochowitz, Frank U Weiss, Matthias Sendler, Markus M Lerch

**Affiliations:** 1 Department of Medicine A, University Medicine, Ernst-Moritz-Arndt-University Greifswald, Greifswald, Germany

## Abstract

Chronic pancreatitis is defined as a continuous or recurrent inflammatory disease of the pancreas characterized by progressive and irreversible morphological changes. It typically causes pain and permanent impairment of pancreatic function. In chronic pancreatitis areas of focal necrosis are followed by perilobular and intralobular fibrosis of the parenchyma, by stone formation in the pancreatic duct, calcifications in the parenchyma as well as the formation of pseudocysts. Late in the course of the disease a progressive loss of endocrine and exocrine function occurs. Despite advances in understanding the pathogenesis no causal treatment for chronic pancreatitis is presently available. Thus, there is a need for well characterized animal models for further investigations that allow translation to the human situation. This review summarizes existing experimental models and distinguishes them according to the type of pathological stimulus used for induction of pancreatitis. There is a special focus on pancreatic duct ligation, repetitive overstimulation with caerulein and chronic alcohol feeding. Secondly, attention is drawn to genetic models that have recently been generated and which mimic features of chronic pancreatitis in man. Each technique will be supplemented with data on the pathophysiological background of the model and their limitations will be discussed.

## Introduction

Chronic pancreatitis is defined as a continuous or recurrent inflammatory disease of the pancreas characterized by progressive and irreversible morphological changes. It typically causes pain and permanent impairment of pancreatic function. In chronic pancreatitis, areas of focal necrosis are typically followed by perilobular and intralobular fibrosis of the parenchyma, by stone formation in the pancreatic duct and by the development of pseudocysts. Late in the course of the disease, a progressive loss of endocrine and exocrine function occurs [1-3].

With an incidence of 8.2, a prevalence of 27.4 per 100 000 population and a frequency of presence in 0.04% to 5% of all autopsies performed, chronic pancreatitis represents a common disorder of the gastrointestinal tract [4-6]. The etiology of this disease is complex and so far a variety of environmental factors including alcohol use, smoking and exposure to other toxic agents as well as endogenous factors such as genetic variations have been described. Chronic pancreatic and biliary tract obstruction or congenital malformations also contribute to development of chronic pancreatitis. Despite advances in the diagnosis of chronic pancreatitis, available treatments are still unsatisfactory because therapeutic concepts are mostly restricted to relieving symptoms rather than changing the natural history [6,7].

### Pathophysiology

The pathogenesis of chronic pancreatitis is still poorly understood. Alcohol use is the leading risk factor and the most common etiology [8]. At present there are four competing hypotheses concerning the pathogenesis of chronic pancreatitis that are largely historic. According to Bordalo *et al*., ethanol induces a fatty degeneration of acinar tissue similar to that seen in hepatocytes during alcoholic liver disease. Ethanol has either a direct or an indirect toxic effect, mediated by the ethanol metabolite acetaldehyde, on the integrity of pancreatic acinar cells [8-10].

Braganza *et al*. proposed a toxic effect of oxygen-derived free radicals on pancreatic acinar cells. Oxidative stress caused by nicotine or ethanol could lead to the peroxidation of the lipid bilayer of the cell membrane, which consecutively disintegrates the membrane [11]. An excess of free oxygen radicals would challenge the protective antioxidant mechanisms, as shown for some cytochrome P450 enzyme pathways in the liver. This hypothesis initiated several clinical studies that tested antioxidants for the treatment of chronic pancreatitis, resulting in some promising observations [12-14]. A large European multicenter study testing the effect of antioxidant treatment in patients with idiopathic chronic pancreatitis is presently recruiting (NCT00142233).

A third hypothesis by Sarles and Sahel proposed a destruction of pancreatic acini due to ductal hypertension resulting from primary intraductal obstruction by protein precipitates as the cause of pancreatitis. This hypothesis asserts that chronic alcohol consumption leads to a decrease in bicarbonate concentration and fluid volume in pancreatic secretions and is therefore associated with the precipitation of protein and calcium crystals within the duct lumen, which ultimately causes duct obstruction. To avoid stone formation, it would be necessary for the acinar cells to produce a low molecular weight protein called lithostatin that would, in turn, increase the fluidity of pancreatic juice and prevent precipitation of protein plaques and calcite crystals in calcium-supersaturated pancreatic juice. The validity of this hypothesis has been questioned because others have failed to confirm a decreased concentration of lithostatins in pancreatic juice from patients with chronic pancreatitis or could not demonstrate an inhibitory function of lithostatins on calcium carbonate precipitation. Nevertheless, the role of duct plugging in cystic fibrosis is unquestioned [15,16]. Klöppel and Maillet revisited the hypothesis of Comfort and colleagues arguing that chronic pancreatitis is a consequence of recurrent episodes of acute pancreatitis [17]. Focal fat necrosis and necrosis of the pancreatic parenchyma would lead to the infiltration of lymphocytes, macrophages and fibroblasts. Fibrosis would thus be assumed to be a consequence of necrosis. This hypothesis would be in accordance with premature intracellular zymogen activation in pancreatic acini as an underlying cause of recurrent bouts of acute pancreatitis, which subsequently lead to the development of chronic pancreatitis. This pathomechanism is also suspected to be the cause of hereditary pancreatitis, which is associated with mutations in the cationic trypsinogen gene [18]. Most of the clinical and experimental evidence suggests that this latter hypothesis predicts the pathophysiology of chronic pancreatitis most accurately.

### Pathophysiology of the development of fibrosis

**Figure 1:**
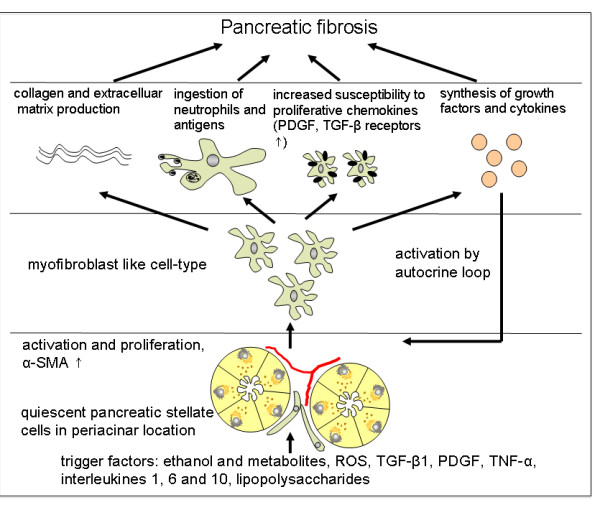
**Role of pancreatic stellate cells (PSCs) in fibrosis**. Activation of PSCs from a quiescent into a myofibroblast-like type and proliferation occurs via several triggers and is perpetuated in an autocrine loop. Development of fibrosis is a complex process that requires interaction of collagen and extracellular matrix production together with chemokine synthesis and phagocytosis.

**Figure 2:**
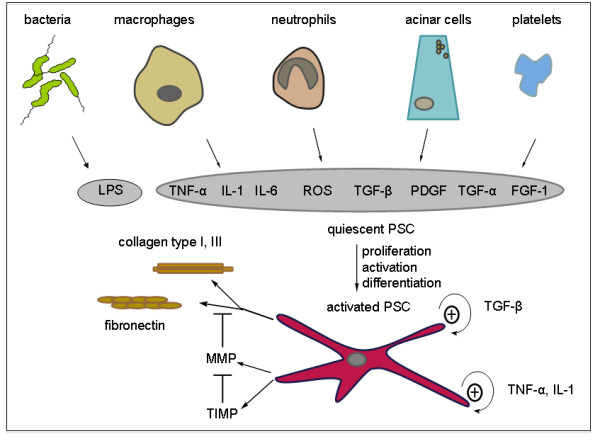
**Multiple activators for pancreatic stellate cells**. Adjacent acinar cells as well as neutrophils, macrophages and platelets stimulate pancreatic stellate cells (PSCs) in a paracrine manner involving growth factors, cytokines and reactive oxygen species (ROS). Bacterial endotoxin lipopolysaccharide (LPS) acts on PSCs. Once activated there is autocrine stimulation perpetuated by transforming growth factor β (TGFβ), tumor necrosis factor α (TNFα) and interleukin 6 (IL-6). They further express extracellular matrix components such as collagens I, III and fibronectin, and matrix metalloproteinases as well as their inhibitors.

Pancreatic fibrosis is a constant histopathological feature of chronic pancreatitis of all etiologies. Fibrosis is generally defined as the accumulation of excessive amounts of extracellular matrix proteins in a tissue. It is now generally accepted that fibrosis is not a mere end product of chronic injury, but an active dynamic process that may be reversible in its early stages. An understanding of the mechanisms responsible for the development of fibrosis has the potential to lead to the development of therapeutic strategies to prevent or retard the fibrotic process. Research into pancreatic fibrogenesis is a rapidly expanding field, one that was given significant impetus by the development of methods to isolate and culture pancreatic stellate cells. On a cellular level and in line with the hypothesis of Klöppel and Maillet, pancreatic fibrosis is caused by activation of pancreatic stellate cells (PSCs) that normally reside in the periacinar region of the pancreas and rest in a quiescent state [19,20]. The role of PSCs is also outlined in Figure 1. They express two cytoskeletal marker proteins, desmin and glial fibrillary acidic protein (GFAP), and can therefore be easily detected by immunostaining and differentiated from fibroblasts. Even if PSCs are quiescent they fulfill an important function for maintenance of synthesis and degradation of the extracellular matrix (ECM) [21,22]. The most crucial step for fibrosis is activation of pancreatic stellate cells, which can occur via different mechanisms (Figure 2). Ethanol and its metabolites, the most important of which is acetaldehyde, cause oxidative stress by reactive oxygen species (ROS) within the gland and finally lead to activation of stellate cells. Secondly, various cytokines such as platelet-derived growth factor (PDGF), transforming growth factor β (TGFβ), tumor necrosis factor α (TNFα) as well as interleukins (IL) 1, 6 and 10 can induce stellate cell activation [22,23]. Third, lipopolysaccharides (LPSs) also activate PSCs by interaction with Toll-like receptor 4 (TLR4) expressed on their surface, indicating a role of endotoxins in the development of pancreatic fibrosis. Notably, alcohol and LPS have a synergistic effect [24]. When pancreatic stellate cells become activated they transform into a myofibroblast-shaped cell type that is capable of migrating easily and secreting increased amounts of extracellular matrix proteins, in particular collagen type I, laminin and fibronectin. Moreover, activated stellate cells can also synthesize cytokines that stimulate PSCs in an autocrine loop [25,26]. Positive staining for α smooth muscle actin (α-SMA) indicates activated stellate cells. There is growing evidence that pancreatic stellate cells act as resident phagocytic cells and, upon activation, they ingest neutrophils and antigens and thus limit the extent of inflammation [27,28]. Progressive fibrosis and destruction of the gland result in exocrine and endocrine insufficiency. These can also be tested for in animal models and are therefore briefly described. Exocrine insufficiency is one sign of chronic pancreatitis, and it normally occurs late in the course of the disease because the pancreas has a great functional reserve. It is defined as either global or partial reduction in the secretion of lipase, amylase or proteases and bicarbonate in the pancreatic juice. Clinical symptoms arise only when lipase secretion is reduced to less than 10% of normal [29]. In clinical practice measurement of exocrine function is performed by either direct or indirect pancreatic function tests. Direct function tests are based on measurement of pancreatic enzymes in the juice that is collected via a duodenal tube, and is considered to be the gold standard for assessing exocrine pancreatic function. However, indirect function tests can detect a decreased amount of pancreatic enzymes in stool or serum or, alternatively, evaluate the digestion of synthetic substrates that are administered orally [30]. The endocrine dysfunction in chronic pancreatitis, also known as pancreatogenic diabetes, manifests with abnormal glucose tolerance and evolves into overt diabetes mellitus. The underlying pathophysiology of diabetes in chronic pancreatitis is mainly based on the loss of insulin secretion. However, there is some evidence that insulin resistance might also contribute to diabetes. Episodes of hypoglycemia can occur and may be lethal due to a lack of countercontrol due to the parallel absence of glucagon [31]. Diabetes mellitus is diagnosed by abnormal glucose tolerance tests (OGTT), alternatively a direct quantification of hypoinsulinemia can be performed by measurements of serum levels of insulin or c-peptide.

### Animal models of pancreatic fibrosis

It should be noted that although a variety of animal models for chronic pancreatitis exist, most of them have been designed to activate pancreatic stellate cells as the principal source of fibrosis. Animal models for studying the development of organ fibrogenesis have been used for many years, and originated in the 1960s when Lieber and DeCarli performed a series of studies investigating the effects of alcohol on the liver. Since then, different animal models have been developed that focus on the effects of alcohol or other pathophysiological stimuli on the pancreas [32,33].

For a systematic presentation of the current animal models, it is useful to group them according to the kind of profibrotic stimulus used (Table 1). Several models combine two or more stimuli to enhance the fibrogenic effects, which makes classification a little more difficult. For the purpose of a clearer presentation we will base our review on the type of trigger employed. The most popular models include ethanol feeding, repetitive caerulein injections and surgical ligation of the pancreatic duct. In addition there are an increasing number of genetic models elucidating the role of one particular gene in the pathogenesis of chronic pancreatitis. Recently, immunological models have attracted more interest, focusing on the role of leukocytes in fibrosis. In the following sections animal models of pancreatic fibrosis will also be assessed according to the extent of fibrosis and organ destruction resulting in exocrine or endocrine insufficiency, and their limitations outlined.

**Table 1:** Animal models for chronic pancreatitis according to the type of stimulus

Model	Stimulus
Duct ligation model	Pancreatic duct ligation
	Incomplete duct ligation
	Occlusion with tissue glue, acrylate, glass particles
Repetitive acute pancreatitis	Serial caerulein injections
	Serial L-arginine injections
Alcohol feeding	Lieber-DeCarli formula
Genetic models	Wistar Bonn/Kobori (WBN/Kob) rats
	R122H transgenic mice
	SPINK3-deficient (SPINK3(-/-)) mice
	CFTR-deficient (cftr^m1UNC^) mice and CFTR(-/-) pigs
	*Kif3a*-deficient mice
	PERK-deficient (PERK(-/-)) mice
	Interleukin 1β transgenic mice (elastase sshIL-1β mice)

CFTR = cystic fibrosis transmembrane conductance regulator; PERK = protein kinase R-like endoplasmic reticulum kinase.

### Pancreatic duct ligation

In 1856 Claude Bernard (1813 to 1878), a student of Francois Magendie, not only reported that pancreatic juice is capable of the emulsification and saponification of lipids and was involved in the digestion of starch and proteins, but also that injection of olive oil into the pancreatic duct of dogs leads to the development of acute pancreatitis. His observations marked the beginning of a century of intensive research into the mechanisms that determine the onset of biliary pancreatitis. The first investigator who systematically addressed the issue of biliary pancreatitis was Eugene Lindsay Opie, who in 1901 published two autopsy reports from which he concluded two mutually exclusive triggering mechanisms for gallstone-induced pancreatitis [34,35]. He employed a series of animal studies to try and support his hypotheses. The first autopsy report found that an impacted gallstone had occluded the orifice of the pancreatic duct and the patient had died from acute pancreatitis [34]. When Opie simulated this finding by pancreatic duct ligation in cats, he noted the development of pancreatic tissue and fat necrosis and proposed pancreatic outflow obstruction as the triggering event for acute pancreatitis. Unfortunately, his first 'impaired outflow hypothesis' was rapidly forgotten after he published his second hypothesis. In another patient who underwent a post mortem examination he found a distinctly different anatomical situation, which he regarded to be of pathophysiological relevance. The impacted stone at the papilla of Vater had created a communication between the common bile duct and the main pancreatic duct, allowing the patient's bile to enter the pancreatic duct. Knowing of the experiments of Claude Bernard, Opie proposed the presence of infected bile in the pancreatic duct as the triggering mechanism of pancreatitis (common channel hypothesis). Despite experimental and clinical evidence that the 'common channel hypothesis' did not explain the pathophysiology of gallstone-induced pancreatitis, it remained popular for more than 75 years. The reasons were (a) the great simplicity with which numerous investigators since Claude Bernard have induced pancreatitis by injecting bile (or any other detergent) into the pancreatic duct, and (b) the bile-stained appearance of necrotic pancreatic tissue that any surgeon who has operated on patients late in the course of necrotizing pancreatitis is aware of. We, and others, have tested Opie's common channel hypothesis in the past, employing the opossum model of acute necrotizing pancreatitis. This model appears ideally suited to test whether bile reflux into the pancreatic duct or blockage of pancreatic secretion triggers pancreatitis [36,37] because the opossum not only possesses a gallbladder, a common bile duct and a single pancreatic duct, but also a long communication between the two. If this common channel is ligated at the papilla of Vater it creates a communication between the pancreatic and bile ducts through which bile could potentially flow [36]. Our experiments showed consistently that neither a common channel, nor reflux of bile into the pancreas is required or likely to be involved in triggering acute necrotizing pancreatitis [37,38].

The most accurate description of the pathophysiology of gallstone pancreatitis is found in Opie's original report, in which he proposes 'pancreatic outflow obstruction' as the most critical event for disease onset. It is therefore probable that pancreatic duct obstruction might also lead to chronic inflammation once the organ is affected by acute injury. However, models for chronic pancreatitis based on duct obstruction are not common and there is only a minority of studies examining the morphological and biochemical changes of the pancreas after duct ligation [39-42]. One of the earliest of these studies was conducted by Churg *et al*. in 1971 in dogs [43]. After ligation of the common bile duct close to the duodenum pancreatic tissue was explanted after 1 week and examined for altered morphology. Architectural changes were distinctive for chronic pancreatitis: pancreata exhibited atrophy of the acinar cells and were characterized by loss of zymogen granules and fragmentation of the rough endoplasmic reticulum (RER). Infiltration of fibroblasts, macrophages and leucocytes was observed in the interstitial space preceding collagen deposition. Luminal widening of the pancreatic ducts was also observed and appeared to be increasing over time after the ligation procedure, partly accompanied with intraductal deposition of cell debris. Apparently the development of pancreatic fibrosis varies among species, as dogs developed later and milder parenchymal changes compared to rats, although the ligation procedures were quite similar. Similar results were observed by Watanabe *et al*., who monitored fibrogenesis for up to 16 weeks after duct ligation [44]. When performing studies in mice the unique anatomy hampers the results: the mouse pancreas consists of three lobes, a gastric, splenic and a duodenal lobe, which drain their juice via individual ducts. In about 70% of cases the splenic duct joins the gastric duct, which finally opens into the common bile duct. There are, unfortunately, variations of this drainage because the anatomy of the mouse pancreas is more complex. Usually the investigator separates the splenic lobe from the rest of the pancreas, consisting of the remaining gastric and duodenal lobe. One of the advantages of this technique is to demonstrate that changes in one distinct part of the organ are indeed caused by mechanical obstruction of the pancreatic duct whereas the rest of the organ remains unaffected and can be used as a control. Secondly the animal does not suffer from a complete loss of organ function. However, this technique is technically challenging as the diameter of the pancreatic duct is only 150 μm and experienced surgical hands are needed to minimize trauma. The pancreatic duct ligation model allows investigation of later effects in the course of chronic pancreatitis. One of the most remarkable observations is the gradual replacement of parenchymal and even fibrotic tissue by fatty tissue after 2 weeks. This phenomenon fits clinical observations in humans and findings at autopsy where atrophy of the exocrine organ is paralleled by fatty tissue replacement [45]. Interestingly, the islets of Langerhans are preserved. In patients suffering from chronic pancreatitis exocrine function is maintained for a long time even in advanced stages of the disease because the functional reserve of the exocrine pancreas is very high and symptoms of exocrine insufficiency only arise when more than 90% of the parenchyma is lost [30,46]. Watanabe *et al*. observed similar results in mice when outflow of about 60% of pancreatic juice was blocked by ligation, but the mice maintained their weight.

The time point and the extent of impairment or even loss of endocrine and exocrine function after duct ligation differ greatly between the studies [47-51]. Glucose intolerance with a concomitant decrease in insulin levels was seen between 28 days and up to 6 to 12 months and there can be a temporary increase of insulin levels shortly after the ligation, which is considered to be a side effect of acute pancreatitis. Exocrine function was predominantly assessed directly by secretin stimulation tests and measurement of bicarbonate and amylase outflow. The limiting factor for comparison of these studies lies in the fact that investigators often combine pancreatic duct ligation with other profibrotic procedures such as ethanol administration, or they only perform an incomplete ligation procedure, as described below. As pancreatic stellate cells are considered to be responsible for the generation of fibrosis, they are expected to become activated upon the proinflammatory stimulus of ligation. α-SMA expression, as an indicator for active pancreatic stellate cells, was detected 7 days after ligation and increased up to the tenth day [52]. Activation of stellate cells is multifactorial and one of the triggers seems to be pancreatic duct obstruction and concomitant elevation of intraductal pressure [51,52].

A modification of the pancreatic duct ligation procedure is incomplete duct obstruction, where the lumen is only reduced by being partially blocked. Technically, a tube (for example, a polyethylene tube) is inserted, resulting in partial obstruction. Critics of the pancreatic duct technique claim that a complete obstruction mainly results in atrophy of the organ and does not necessarily lead to the typical morphology of chronic pancreatitis. In contrast, incomplete obstruction would produce milder but more typical alterations of the tissue architecture [47]. In an alternative to ligation, the pancreatic duct can be occluded by tissue glues such as acrylate or prolamine or by glass particles, resulting in similar changes to that described above [53,54].

### Caerulein-induced pancreatitis

It is generally believed that the morphological changes that characterize acute pancreatitis result from digestion of the gland by enzymes that are normally synthesized and secreted by pancreatic acinar cells [36,55,56]. Evidence that support this notion include the observations that (a) the morphological changes of severe pancreatitis resemble those that are typical of digestive necrosis [57]; (b) pancreatic acinar cells synthesize digestive enzymes, which when activated, lead to digestive necrosis of the gland [58]; and (c) activated digestive enzymes have been detected within the gland during severe pancreatitis [59]. Most of the potentially harmful digestive proteases of acinar cells are normally synthesized and secreted as inactive zymogens and are activated only in the duodenum by brushborder enzymes [60]. As early as in 1895 Mouret *et al*. reported that excessive cholinergic stimulation is associated with the development of pancreatic injury determined by acinar cell vacuolization and necrosis [61]. Mouret *et al*. suggested that the activation of trypsin might be actively involved in the development of acute pancreatitis. This hypothesis was in accordance with the observations of Hans Chiari, who proposed autodigestion to be the pathomechanism underlying acute pancreatitis in 1896 [55]. Subsequently, experimental animal models employing cholinergic agonists such as carbamylcholine and charbachol, cholecystokinin (CCK) and its analogs, as well as scorpion venom were shown to induce pancreatic injury in a manner both time and dose dependent [62-66]. In rodents CCK plays a major role in regulating exocrine pancreatic secretion after stimulation by food ingestion. However, human pancreatic acinar cells may not respond directly to CCK stimulation but are mostly regulated by cholinergic pathways that involve neurogenic CCK stimulation [67]. In 1977 Lampel and Kern characterized the clinical and biochemical patterns of acute interstitial pancreatitis in rats after administration of excessive doses of pancreatic secretagogue [68]. The most prominent morphological characteristic is the development of excessive edema as early as 1 h after the onset of the disease [69]. Since that time the model of pancreatitis induced by caerulein (a CCK analog derived from the Australian tree frog *Litoria caerulea*) in rodents is widely used and one of the best characterized experimental varieties.

The primary physiological effect of CCK and its analogs on the pancreas is to stimulate protein-rich secretion; it has a lesser effect on fluid and electrolyte secretion. Doses of CCK that lead to continued maximal stimulation of enzyme secretion are associated with increased rates of both protein synthesis and the movement of newly synthesized proteins through the secretory pathway. The increase in protein synthesis is outpaced by the rate of protein secretion. Thus, following stimulation with maximal secretory doses of caerulein, the enzyme stores of the exocrine pancreas may be reduced by as much as 75% within several hours. Increasing the concentration of CCK by an order of magnitude over the levels that produce maximal secretion is known as supraoptimal stimulation, supramaximal stimulation or hyperstimulation [69]. Compared to maximal stimulation, supramaximal stimulation generates a distinct pancreatic response that includes diminished secretion, accumulation of secretory proteins within the pancreas and pancreatic injury. The route of administration for caerulein that induces acute pancreatitis differs in various rodents, as does the severity of the disease [70-73]. While in rats caerulein can be continuously infused intravenously either via a polyethylene catheter placed into the external jugular vein or into the tail vein, in mice caerulein is generally injected repeatedly into the peritoneal cavity [74]. The caerulein concentration that results in pancreatic edema, increased serum levels of pancreatic enzymes, inflammation and necrosis ranges between 5 to 10 μg/kg/h in rats and thereby exceeds the maximal secretory concentration 10-fold to 20-fold. Maximal pancreatic injury occurs after 12 h of continuous infusion but changes can be monitored already 15 minutes after the start of the caerulein infusion and resolve spontaneously after 24 to 48 h. One of the earliest consequences of hyperstimulation is the formation of pancreatic edema. This increase in pancreatic fluid, which occurs within the first hour of caerulein hyperstimulation, is probably the result of several factors: increased vascular permeability, increased hydrostatic pressure from the constriction of small vessels and increased tissue oncotic pressure from the interstitial release of pancreatic enzymes and hydrolytic products. Under the conditions of supramaximal caerulein stimulation, secretion of zymogens into the pancreatic duct is virtually abolished and premature zymogen activation can be observed after a sustained intracellular calcium rise and a breakdown of the actin cytoskeleton. These events lead to a systemic inflammatory response syndrome, which includes extrapancreatic damage such as pancreatitis related lung injury.

One of the most frequently used methods to induce chronic fibrotic changes in the pancreas is to perform self-limited acinar cell injury by repeated pathological stimuli of the gland. The kind of stimulating agent and the intervals are variable. This method mimics the clinical observation that repeated episodes of acute pancreatitis, irrespective of its origin, lead to increasing damage of the organ that eventually results in atrophy and fibrosis [75]. At the beginning of the recovery period the pancreas produces components of extracellular matrix that temporarily exceed the degradation of the extracellular proteins [20]. Secondly, profibrotic cytokines are released leading to an environment that favors fibrosis. One of the most potent fibrogenic modulators is TGFβ1, which is overexpressed in pancreatic acinar and stromal cells after caerulein-induced pancreatitis [76,77]. In this phase the organ is extremely vulnerable to repeated episodes of acute pancreatitis and will not be able to degrade the ECM components, which finally promotes fibrosis after a number of repeated injuries [78]. Neuschwander-Tetri *et al*. performed injections twice a week for 10 weeks in mice and could observe prominent fibrotic changes of the pancreas. Due to the dysbalance between accumulation and degradation of extracellular matrix in this experimental set-up, an increased collagen production started around the second week and mainly consisted of collagen type I deposited in the periacinar region. In regions with severe fibrosis, acinar cells displayed features of ductal cells with loss of zymogen granules and a more centrally located nucleus. Since they also surround the lumen of tubular structures they were termed 'tubular complexes' that are typically found in chronic pancreatitis [79,80].

Repetitive caerulein injections alone only cause minor effects on endocrine cells as shown in studies performed in rats. Since metabolic impairment normally occurs late in the course of the disease animals were challenged by a second stimulus to accelerate development of chronic pancreatitis. In one model water immersion stress was used, which provokes repeated tissue ischemia and reperfusion. Here, rats showed a diabetic pattern with elevated blood sugar and reduced insulin levels [81,82]. These findings also led to the assumption that caerulein injections alone were not sufficient to provoke endocrine dysfunction and that toxins have to be used to increase the severity of chronic pancreatitis. Data on exocrine insufficiency in an animal model of repetitive caerulein injections are rare. According to the observations of Ohashi *et al*. deficiency in exocrine function can occur after 6 weeks by significant decrease of pancreatic protein content that was used as an indicator for exocrine function [83]. Wistar Bonn/Kobori (WBN/Kob) rats with chronic pancreatitis that sequentially underwent intravenous applications of caerulein showed more severely impaired dysfunction assessed by cholecystokinin-stimulated flow rate, bicarbonate output, and protein output compared to untreated rats [84]. However it has to be considered that WBN/Kob rats themselves already develop chronic pancreatitis even if there is no additional trigger for pancreatitis. In summary, exocrine pancreatic insufficiency can be achieved in a caerulein model, but for endocrine insufficiency more than one trigger or predisposing factor for pancreatitis is required.

There are many variations regarding the amount and frequency of caerulein injections and extension of fibrosis is highly variable [83,85,86]. Studies using lower caerulein concentrations or fewer injections such as only once a week or even every third week have shown that architectural alterations of the pancreas develop later and occur as late as 1 year after onset of injections. It is likely that there is a dose dependency for caerulein, a dependency on the injection intervals and the extension of pancreatic damage. Using higher frequencies of caerulein applications will lead to rapid formation of pancreatic fibrosis.

### The l-arginine model

The essential amino acid L-arginine has been shown to cause acute pancreatitis in murine and rat models [87-89]. With the aim to establish a non-invasive animal model burdened with a significant mortality, in 1984 Mizunuma *et al*. developed a new type of experimental pancreatitis by intraperitoneal administration of high concentrations of L-arginine in rats [90]. In subsequent studies it was shown that L-arginine leads, in a dose dependent manner, to acinar cell necrosis levels of up to 100%. At 24 h after the first intraperitoneal injection the pancreas doubles its weight and ultrastructural examinations reveal partial distension of the endoplasmic reticulum. At 48 h after the onset of pancreatitis dissociation and necrosis of acinar cells was noted. Subsequently, necrotic cells are replaced by interstitial tissue composed of leukocytes and fibroblasts [91,92]. The mechanism by which L-arginine causes pancreatitis is not fully understood. Furthermore, the crucial question of whether excessive concentrations applied intraperitoneally cause premature intracellular zymogen activation is not answered. Several reports suggest that oxygen free radicals, nitric oxide and inflammatory mediators might play a key role. Long-term administration of L-arginine for 30 days induces pancreatic atrophy, with exocrine pancreatic insufficiency resembling the clinical picture of chronic pancreatitis [93]. Histological changes appear slightly later than in caerulein-induced pancreatitis (40 days in arginine pancreatitis compared to 35 days in caerulein-treated animals) and morphological changes mainly consist of collagen IV depositions around acini, vessels and ducts. After 2 months normal pancreatic parenchyma was replaced by fatty tissue and ducts showed caliber dilations [94]. There are even studies indicating that fibrotic changes can appear as early as 1 week after onset [92].

### Combination of repetitive caerulein injections with toxins and other agents

There are several reports showing that the combination of serial caerulein injections together with other proinflammatory agents enhances pancreatic fibrogenesis. 

Table 2

Toxic agents frequently used in addition to serial caerulein injections

Lipopolysaccharides (LPS)

Cyclosporin A (CsA)

Dibutyltin dichloride (DBTC)

Ethanol

**Figure 3:**
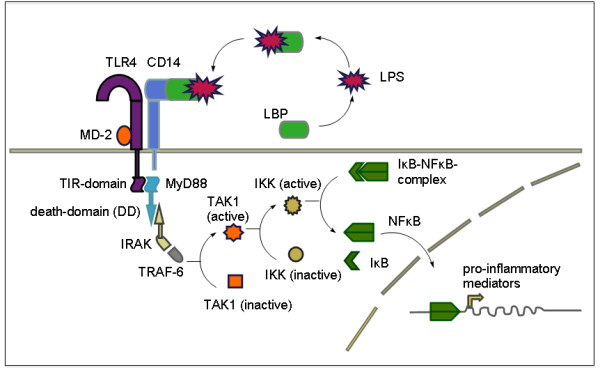
**Lipopolysaccharide (LPS)-dependent and nuclear factor (NF)κB-mediated activation of proinflammatory cytokines**. LPS binds to LPS-binding protein (LBP) and forms a complex with the surface protein CD14 that further interacts with MD-2 associated Toll-like receptor 4 (TLR4). Myeloid differentiation primary response gene 88 (MyD88) is recruited to TLR4 by its Toll-interleukin 1 receptor (IL1R) (TIR) domain and further activates serine/threonine kinase interleukin 1 receptor-associated kinase (IRAK) by its death domain (DD). After recruitment of tumor necrosis factor receptor associated factor 6 (TRAF-6), another adapter protein, mitogen-activated protein kinase kinase kinase (MAPKKK) transforming growth factor β activated kinase (TAK1) is activated that phosphorylates the inhibitor of κB kinase (IKK) complex. Activated IKK removes inhibitory IκB from the IκB-NFκB complex so that NFκB can enter the nucleus and where it stimulates the expression of proinflammatory chemokines. In a second pathway, which is not listed here, TAK1 is able to activate MAP kinases p38 and c-Jun N-terminal kinase (JNK).

Secondly, intraperitoneal caerulein injections can also be administered in genetically transformed mice to enhance the effects. Due to its ease of use the repetitive caerulein model has evolved to be a popular method for generation of pancreatic fibrosis. LPS is the major component of the outer membrane of Gram-negative bacteria, contributing greatly to the structural integrity of the bacteria, and protecting the membrane from chemical attack. LPS also increases the negative charge of the cell membrane and helps stabilize the overall membrane structure. It is of crucial importance to Gram-negative bacteria, whose death results if it is mutated or removed. LPS is an endotoxin, which induces a strong immune response in animals (Figure 3). When attached to LPS binding protein (LBP), an acute phase protein, this complex binds to CD14, which further activates TLR4. TLR4 mediates an intracellular signaling cascade involving the adapter protein myeloid differentiation primary response gene 88 (MyD88) and serine/threonine kinases leading finally to a dissociation of the IκB/nuclear factor (NF)κB complex [95-99]. This dissolution is a prerequisite for activation of NFkB, which enters the nucleus and stimulates synthesis of proinflammatory cytokines in monocytes and macrophages [95,100]. Recently it was shown that, as well as in immune cells, pancreatic stellate cells also express CD14 and TLR4, suggesting a direct effect of LPS on PSCs [24]. LPS aggravates acute pancreatitis when applied intraperitoneally or intravenously [101]. LPS also accelerates the development of chronic pancreatitis as shown by Ohashi *et al*. In this study the effect of caerulein alone and the combination of LPS with caerulein in mice was monitored [83].

Table 2

Toxic agents frequently used in addition to serial caerulein injections

Lipopolysaccharides (LPS)

Cyclosporin A (CsA)

Dibutyltin dichloride (DBTC)

Ethanol

**Figure 4:**
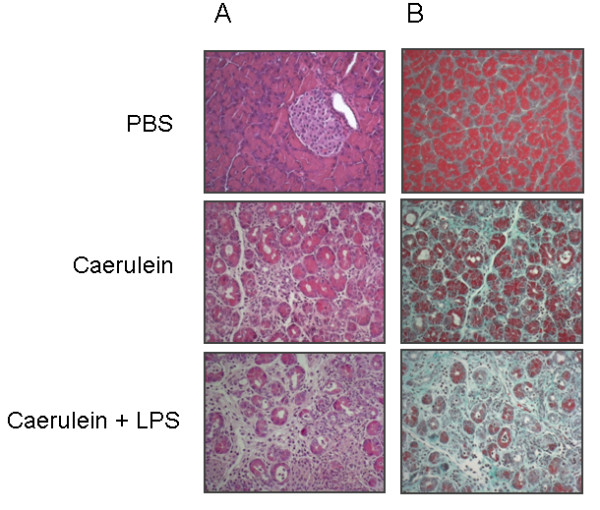
**Histological changes of pancreatic tissue architecture after repetitive caerulein and combined caerulein-lipopolysaccharide (LPS) injections**. **(A) **Hematoxylin and eosin staining indicates destruction of pancreatic acinar cells and development of tubular complexes, a sign of chronic pancreatitis. **(B) **Masson-Goldner trichrome stain denotes fibrosis; connective tissue is stained green. Caerulein and LPS increase pancreatic fibrosis compared to caerulein alone. Phosphate-buffered saline (PBS)-treated mice did not develop chronic pancreatitis and were used as negative controls.

Results from our group confirm that the addition of LPS to caerulein increases destruction of pancreatic acini. Mice were treated with caerulein (50 μg/kg body weight) or both caerulein and LPS (3,125 mg/animal) twice a week for 10 weeks. Thereafter mice were killed and pancreata were subjected for histological analysis. Both groups showed acinar cell atrophy, invasion of leucocytes and formation of tubular complexes. Pancreata of mice that were treated with caerulein and LPS had more severe signs of chronic pancreatitis (Figure 4A). Masson-Goldner trichrome stains visualized the extension of fibrosis that was enhanced after addition of LPS (Figure 4B). Control animals that were treated with PBS alone did not show any signs of chronic pancreatitis. Lipopolysaccharides are toxic and animals die from anaphylaxis after exposure to LPS. Therefore dose-response experiments for determination of the right LPS dose are needed [102]. Cyclosporin A (CsA) is a widely used immunosuppressant used after organ transplantation and one of its mode of action is to induce TGFβ expression. Due to the effect of TGFβ on pancreatic stellate cells and its capability to inactivate collagenases CsA shifts the balance to a profibrogenic state. Cyclosporin A treatment alone temporarily raised TGFβ blood levels but pancreatic morphology was mostly conserved and did not show characteristic signs of chronic pancreatitis. In combination with caerulein injections prominent histological damage with increased collagen deposition and glandular atrophy was observed, which exceeded the effects of caerulein alone. Moreover CsA is also able to reduce pancreatic blood flow and thus potentiates the damage to the gland [103]. In a model termed the 'cyclosporin A model of alcoholic chronic pancreatitis' by Gukovsky *et al*. addition of CsA to caerulein in rats that were additionally fed with ethanol led to a decrease of pancreatic parenchyma by approximately 86% and a severe impairment of the functional reserve. In particular, ethanol feeding was responsible for extensive fibrosis and impaired regeneration [104]. One of the limitations of cyclosporin A treatment is that it accumulates in the pancreas and can directly cause morphological and functional changes to the organ such as cellular atrophy and a decrease in exocrine function. These changes are concentration dependent and therefore CsA needs to be titrated to avoid local toxic effects [105].

Induction of toxic damage to the pancreas can be achieved by dibutyltin dichloride (DBTC), a compound that belongs to the chemical class of organotins that are industrially used for heat stabilization of polyvinylchloride plastics, as biocides in agriculture, and by shipbuilders as antifouling paint. The twofold effects of DBTC on the pancreas are mediated by a biliary obstructive component and secondly a direct toxic effect. Organotins are rapidly excreted via the bile and cause damage to the bile duct epithelium with subsequent necrosis and duct obstruction. Due to cholestasis, biliary pancreatitis and pancreatic necrosis develop. The second effect of DBTC is mediated by its direct toxic effect on acinar cells, mediated via hematogenic transport to the gland. Repeated intravenous applications of DBTC at intervals of 3 weeks induce acute interstitial pancreatitis in the first week and later interstitial and periductular fibrosis which are detectable after 6 weeks [106-108]. It has to be taken into account that this model also induced severe damage to the liver including bile duct hyperplasia, inflammatory cell invasion and parenchymal necrosis. Dibutyltin dichloride has a half-life of about 2 days, and there is a dose-dependent relation of DBTC and the toxic effects on the pancreas and the liver. Due to its dependence on bile duct anatomy this model is only applicable in rats.

### Inhibitory factors for development of pancreatic fibrosis

In line with the pathophysiological concept put forward by Joan Braganza, oxidative stress caused by ROS is known to be proinflammatory and favors acute and chronic pancreatitis by direct damage of cells as well as by activation of inflammatory cells and pancreatic stellate cells [109]. Overexpression of thioredoxin (TRX-1), which is an endogenous anti-inflammatory and antioxidative protein attenuated pancreatic fibrosis in a model of repeated caerulein injections in TRX-1 transgenic mice. Monocyte chemotactic protein 1 (MCP-1), a very potent attractant for inflammatory cells that further synthesize profibrotic chemokines, was significantly reduced in this model. Furthermore, exocrine function was preserved [83]. TGFβ is a pleiotropic cytokine consisting of different isoforms with an anti-inflammatory effect via suppression several proinflammatory cytokines such as interferon γ (IFNγ), various interleukins, and T and B cells as well as macrophages. However, TGFβ can also be profibrogenic as it activates stellate cells and thus promotes fibrosis in different organs such as the liver and the pancreas. Inhibition of the TGFβ signaling pathway ameliorated pancreatic fibrosis. A dominant-negative mutant of the TGFβ receptor or overexpression of Smad7, an intracellular protein that negatively regulates TGFβ signaling results in significantly reduced fibrosis in experimental pancreatitis models [110,111].

### Repeated alcohol feeding

Alcohol use is one of the main causes of acute and chronic pancreatitis in Western countries but there is still much controversy about how chronic alcohol consumption leads to chronic pancreatitis. About 70% of chronic pancreatitis cases are associated with alcohol abuse but less than 10% of chronic alcoholics develop chronic pancreatitis [75,112].

Alcoholic-induced pancreatic damage is thought to be caused by different effects of ethanol and its metabolites. In the liver alcohol can be metabolized, whereas in the pancreas the relevant enzymes necessary for metabolism are not expressed [113,114]. Thus toxic compounds exert different effects on the pancreas. ROS are byproducts of ethanol metabolism and have a direct toxic effect on pancreatic acinar cells. Secondly, pancreatic stellate cells as the major source of fibrosis are activated by ROS [21]. In isolated acini of ethanol-fed animals trypsin and chymotrypsin activation was markedly increased upon cholecystokinin stimulation compared to control animals [115].

Due to the high impact of ethanol on pancreatic diseases alcohol was frequently used as a trigger for chronic pancreatitis in animal models, and the first animal models for chronic pancreatitis ever developed were based on alcohol feeding. One of the earliest studies investigating the effects of ethanol administration on different organs was published by Lieber and DeCarli, who repeatedly fed rats and baboons with ethanol as part of their diet [116,117]. The animals developed fatty liver disease, alcoholic hepatitis and later on cirrhosis. The feeding regime, which was named the 'Lieber-DeCarli Formula', was based on supplementation of ethanol to a liquid diet and allows an increased total alcohol intake compared to that possible with pure ethanol feeding alone. It has been shown to be one of the most useful methods to imitate the effects of alcohol on various organs. In 1971 Sarles *et al*. observed histological changes in the pancreas comparable to chronic pancreatitis in more than 50% of Wistar rats after chronic intake of ethanol for 20 to 30 months [118]. The duration of ethanol feeding differs among studies, and mostly varies between 4 weeks and 16 months although longer observation times have been reported [112]. It is well documented that ethanol leads to severe organ damage in the liver but pancreatic morphology changes are milder; rather than chronic pancreatitis only mild functional insufficiency develops in most cases. These observations correlate with the clinical finding that only a minority of alcoholics ever develop chronic pancreatitis despite regular immoderate alcohol consumption. Ethanol supplementation to daily food for a total of 6 months in rats resulted in exocrine impairment but not in morphological changes characteristic for chronic pancreatitis [112]. Even administration by continuous intragastric infusion could not cause chronic injury to the pancreas of rats. It is therefore still controversial whether a satisfactory model for chronic pancreatitis induced by ethanol alone is feasible, and it is more likely that ethanol has to be regarded as a cofactor for the development of fibrosis in 'preinjured' animals. Moreover, alcohol diet or infusions not only cause damage in the pancreas but also to many other organs such as the liver, kidneys and lungs, which has to be taken into account when studying systemic effects [32,118,119].

The combination of alcohol feeding with caerulein injections exacerbates the course of pancreatitis and increases pancreatic fibrosis and loss of parenchyma. Additionally, calcifications indicating severe chronic pancreatitis can be observed. Data on functional impairment are sparse but there is evidence that digestive enzyme synthesis is reduced after chronic ethanol feeding [104,120-122]. All in all, the majority of studies indicate that ethanol administration alone only causes few, if any, changes that are typical for chronic pancreatitis, which implies that alcohol mainly serves as a sensitizer to chronic injury.

### Hereditary animal models on chronic pancreatitis

Chronic pancreatitis is a complex inflammatory disease and over the past decade there have been increasing efforts focusing on genetic abnormalities that predispose people to chronic pancreatitis. Genetic analyses can identify pancreas-specific factors that are associated with higher or lower susceptibility to acute or chronic pancreatitis. Activation of trypsinogen is one of the key events in the early phase of pancreatitis and therefore genetic abnormalities found in the trypsinogen gene and in its inhibitors might be of particular importance. In addition, much attention is focused on cystic fibrosis, a hereditary disease of chloride ion channels (cystic fibrosis transmembrane conductance regulator (CFTR)) accompanied by pancreatic fibrosis together with functional defects of the lungs and the small intestine. However, there are some other genetic models that might help us to understand how chronic pancreatitis develops, and those will be outlined below.

### WBN/Kob rats

Male WBN/Kob rats endogenously develop chronic pancreatitis-like lesions. This strain, originating from a Wistar rat colony at the University of Basel, was generated at the University of Bonn in 1961 by brother-sister inbreeding and was primarily reported by Kobori *et al*. to be susceptible to tumor induction in the glandular stomach [123,124]. The first changes were described after 3 months of age, starting with periductular fibrosis leading to extensive parenchymal loss and fibrosis progressing rapidly with age. Exocrine and endocrine function was also impaired as demonstrated by reduced excretion of *N*-benzoyl-L-tyrosyl-*p*-aminobenzoic acid (NBT-PABA) in the urine and glycosuria [125]. Eventually WBN/Kob rats become diabetic at 60 to 90 weeks of age. Thus WBN/Kob rats emerged as a useful animal model for studying chronic pancreatitis because they show both morphological and biochemical changes typical of this disease. So far the underlying mechanism is incompletely understood, and several possibilities are being discussed. Chronic pancreatitis only occurs in male rats around the time when the animals become sexually mature and androgen synthesis is increased. Furthermore, estradiol treatment ameliorates diabetic symptoms whereas ovariectomy causes pancreatic fibrosis in females. These findings suggest that sex hormones are involved in the development of chronic pancreatitis. Enhanced apoptosis of acinar cells was found in WBN/Kob rats preceding the infiltration of inflammatory cells and was reversible after glucocorticoid treatment, suggesting that programmed cell death might be of importance for the loss of parenchymal cells. Mitochondrial swelling after ischemia and stasis of pancreatic juice were further reported to be responsible for early pancreatic changes [125,126]. Chromosomal mapping of WBN/Kob rats showed a unique haplotype block in the chromosomal region Pdwk1 (pancreatitis and diabetes mellitus in WBN/Kob locus 1) on chromosome 7 after identification of nucleotide polymorphisms of three candidate genes that were not found in other inbred rat strains [127]. These genes (*Rac2*, *Grap2 *and *Xpnpep3*) exert different functions. *Rac2 *encodes for a GTPase that belongs to the RAS superfamily and is involved in diverse cellular events including apoptosis and phagocytosis. *Grap2 *is an adaptor protein involved in leukocyte specific protein tyrosine kinase signaling, whereas *Xpnpep3 *is a mitochondrial protein important for proper ciliary function [128]. Clearly, genetic alterations play a role in pancreatic fibrosis of this rat strain.

### Transgenic expression of R122H trypsinogen

The premature trypsinogen activation within the pancreatic parenchyma is a crucial event for the initiation of pancreatitis. Once activated, trypsin is able to activate other digestive proenzymes in the pancreas. In 1996 Whitcomb *et al*. reported on arginine-histidine mutation (R122H) in human cationic trypsinogen (PRSS1) that is associated with an autosomal dominant, hereditary pancreatitis phenotype; a rare type of pancreatitis characterized by chronic inflammation and necrosis [129]. A transgenic mouse model carrying the missense mutation R122H in murine trypsin 4 displayed pancreatic fibrosis and acinar cell dedifferentiation with progressing age. Another model using R122H mutated human trypsinogen, which was expressed under control of a rat elastase-2 promoter in the mouse pancreas, showed elevated levels of lipase but no spontaneous development of chronic pancreatitis. Both mouse models were more susceptible to caerulein-induced pancreatitis developing more severe pancreatitis underscoring the importance PRSS1 mutations as a pathogenic mediator [130,131]. Interestingly, in a recently published model by Gaiser and coworkers intra-acinar expression of active trypsin *in vivo *was sufficient to induce acinar death and local and systemic inflammation. Of note, intra-acinar active trypsin led to acinar cell death by both necrosis and apoptosis *in vivo*. Remarkably, sustained intra-acinar trypsin activity resulting from repeated tamoxifen administration every fifth day over 40 days led to massive acinar cell loss caused by ongoing cell death and significant fatty replacement was observed. However, there was no evidence of chronic inflammation or of fibrosis; both hallmarks of chronic pancreatitis [132].

Mutations in other genes such as anionic trypsinogen (PRSS2), the serine protease inhibitor Kazal type 1 (SPINK1), CFTR, chymotrypsinogen C (CTRC) and calcium-sensing receptor (CASR) are also associated with an increased risk for pancreatitis [133,134]. A knockout model of murine SPINK3, which is a homologue of human SPINK1, is lethal within 2 weeks after birth. At around 16.5 days after coitus acinar cells showed autophagic degeneration with no signs of parenchymal regeneration. Enhanced trypsin activity was detected in isolated acini [135,136]. Targeted overexpression of pancreatic secretory trypsin inhibitor I increased endogenous trypsin inhibitor capacity by 190% in transgenic compared to wild-type mice whereas trypsinogen activation remained constant. Severity of pancreatitis was decreased in these transgenic mice [137].

### Cystic fibrosis

Cystic fibrosis (CF) is a common autosomal recessive disease with a carrier rate of about 5% in Caucasians. In 1989 the underlying gene was found, coding for a chloride channel that was named CFTR. Subsequently, more than 1,000 gene mutations associated with cystic fibrosis have been reported. The defective chloride channel causes abnormal chloride transport through epithelial cells leading to deficient fluid secretion and thickening of secretions not only in the pancreas but also in the respiratory airways, kidneys and intestine [138]. Pancreatic excretory function is diminished in up to 90% of patients with CF, and shows wide variations ranging from complete loss of pancreatic function up to an almost normal phenotype [134,139].

Mice homozygous for the disrupted CFTR gene (cftrm1UNC) display many features of cystic fibrosis patients such as meconium ileus, alterations of mucous and serous glands and obstruction of glandular ducts by inspissated material. Death occurs after around 40 days of age by intestinal obstruction and ileus [140]. The exocrine pancreas of the CFTR knockout mouse is morphologically altered with fewer zymogen granules and partly dilated ducts filled with amylase-positive aggregates and lined by high amounts of sulfated glycoproteins. The duodenal pH of the CF mice was shifted to more acidic condition, explained by decreased bicarbonate production. Mild exocrine insufficiency was observed in CFTR(-/-) mice that had less pancreatic enzyme secretion and lower protein and mRNA levels of digestive enzymes. Mice developed more severe acute pancreatitis after caerulein hyperstimulation, which was explained by a decrease of apoptosis and a higher baseline proinflammatory state indicated by constitutively higher expression of inflammatory cytokines [141,142]. In order to establish a model that is closer to humans regarding lifespan, genetics and physiology Rogers *et al*. recently developed a porcine model with a CFTR defect [143]. Morphological changes were more distinct than those described above and were similar to the findings in newborn patients with cystic fibrosis. The limiting factor of this model is, besides the more costly handling of animals, that all piglets develop meconium ileus with a high risk of perforation and peritonitis. Therefore they require surgery to resolve intestinal obstruction. The overall survival of animals is less due to perioperative and postoperative complications along with a failure to thrive.

### Other genetic models

Defects in cilia formation are associated with several genetic diseases including polycystic kidney disease and primary ciliary dyskinesia, also known as Kartagener syndrome, that affects the cilia of the respiratory tract. Absence of cilia in pancreatic epithelial cells produces lesions that resemble chronic pancreatitis tissue. By conditional inactivation of the *Kif3a *gene, encoding a subunit of the kinesin-2 complex that is essential for cilia formation fibrosis, lipomatosis and cyst formation was observed in pancreatic tissue [144,145]. Disruption of proper protein folding in the endoplasmic reticulum (ER) results in accumulation of misfolded proteins and finally ends up with altered gene expression and ER stress. Protein kinase R-like endoplasmic reticulum kinase (PERK), a transmembrane molecule of the endoplasmic reticulum, attenuates translation in response to ER stress and absence of PERK renders cells to be more susceptible to agents causing ER stress and protein misfolding [146]. PERK(-/-) mice rapidly experience a decline of endocrine and exocrine pancreatic function, observed between 4 and 8 weeks of age, which could be attributed to increased apoptosis of insulin-producing and acinar cells. Recently the importance of cytokines was pointed out in a transgenic mouse model overexpressing human IL-1β in the pancreas. This cytokine has been shown to be involved in acute pancreatitis and pathogenesis of multisystem organ failure. Elastase sshIL-1β mice consistently developed severe chronic pancreatitis similar to human disease [147,148]. In this model propagation of more proinflammatory cytokines and recruitment of leucocytes triggered by IL-1β are considered to be the underlying causes for fibrosis. Another mechanism is a direct activation of stellate cells and T lymphocytes that exert a cytotoxic effect on pancreatic cells [149]. The authors pointed out that the severity of chronic pancreatitis correlated with the expression of IL-1β. Compared to mice that were treated by serial caerulein injections for 20 weeks sshIL-1β mice of the same age had more severe signs of chronic inflammation. No significant differences regarding pancreatic exocrine and endocrine function were detected. Taken together, these findings underline the importance of immune activation for chronic inflammation; secondly, the model might be a tool for investigation of carcinogenesis in chronic pancreatitis as older IL-1β transgenic mice display acinar-ductal metaplasia.

## Conclusions

The intention of this review was to describe the most frequently used and best established models for chronic pancreatitis in animals. Most are rodent models, since mice and rats are easy to handle and there is a steadily increasing number of genetic models obtained by gene deletion or transgenic expression of genetic variants. Choosing the right model is difficult and the scientific rationale needs to be carefully considered. Secondly, not all models of chronic pancreatitis parallel all classical symptoms and the question addressed is of importance when choosing a model. Repetitive caerulein injections are amongst the most widely used models. Technically, caerulein injections are relatively easy to perform and they show a high reliability and reproducibility. Another advantage is that other compounds mediating injury such as lipopolysaccharides or cyclosporin A can be easily added. Third, serial caerulein injections can be performed in transgenic or knockout animals and will allow future studies to answer how different predispositions exert additive effects or abrogate each other, depending on the gene that is overexpressed or deleted. Established protocols notwithstanding, the right dose and the optimal injection interval have to be established in individual laboratory tests before each set of experiments.

## Competing interests

The authors declare that they have no competing interests.

## Authors' contributions

AAA and JM designed and drafted the manuscript. SC made significant contributions to the design of the figures and provided experimental data. FUW and MS gave their intellectual input to the manuscript. MML drafted the initial outline of the manuscript and finalized the content. All authors read and approved the final manuscript.

## References

[B1] Ammann RW Akovbiantz A Largiader F Schueler G Course and outcome of chronic pancreatitis. Longitudinal study of a mixed medical-surgical series of 245 patients Gastroenterology 1984 86 820 828 6706066

[B2] Etemad B Whitcomb DC Chronic pancreatitis: diagnosis, classification, and new genetic developments Gastroenterology 2001 120 682 707 10.1053/gast.2001.22586 11179244

[B3] Sarner M Cotton PB Classification of pancreatitis Gut 1984 25 756 759 10.1136/gut.25.7.756 6735257 PMC1432589

[B4] Chari ST Singer MV The problem of classification and staging of chronic pancreatitis. Proposals based on current knowledge of its natural history Scand J Gastroenterol 1994 29 949 960 10.3109/00365529409094869 7839103

[B5] Andersen BN Pedersen NT Scheel J Worning H Incidence of alcoholic chronic pancreatitis in Copenhagen Scand J Gastroenterol 1982 17 247 252 10.3109/00365528209182047 7134849

[B6] Olsen TS Lipomatosis of the pancreas in autopsy material and its relation to age and overweight Acta Pathol Microbiol Scand A 1978 86A 367 373 716899 10.1111/j.1699-0463.1978.tb02058.x

[B7] O'Sullivan JN Nobrega FT Morlock CG Brown AL Jr Bartholomew LG Acute and chronic pancreatitis in Rochester, Minnesota, 1940 to 1969 Gastroenterology 1972 62 373 379 5011528

[B8] Lowenfels AB Maisonneuve P Cavallini G Ammann RW Lankisch PG Andersen JR DiMagno EP Andren-Sandberg A Domellof L Di Francesco V Prognosis of chronic pancreatitis: an international multicenter study. International Pancreatitis Study Group Am J Gastroenterol 1994 89 1467 1471 8079921

[B9] Ammann RW Heitz PU Kloppel G Course of alcoholic chronic pancreatitis: a prospective clinicomorphological long-term study Gastroenterology 1996 111 224 231 10.1053/gast.1996.v111.pm8698203 8698203

[B10] Bordalo O Goncalves D Noronha M Cristina ML Salgadinho A Dreiling DA Newer concept for the pathogenesis of chronic alcoholic pancreatitis Am J Gastroenterol 1977 68 278 285 596358

[B11] Schoenberg MH Buchler M Pietrzyk C Uhl W Birk D Eisele S Marzinzig M Beger HG Lipid peroxidation and glutathione metabolism in chronic pancreatitis Pancreas 1995 10 36 43 10.1097/00006676-199501000-00005 7899458

[B12] Braganza JM Pancreatic disease: a casualty of hepatic "detoxification"? Lancet 1983 2 1000 1003 6138545 10.1016/s0140-6736(83)90983-2

[B13] Braganza JM Wickens DG Cawood P Dormandy TL Lipid-peroxidation (free-radicaloxidation) products in bile from patients with pancreatic disease Lancet 1983 2 375 379 6135876 10.1016/s0140-6736(83)90347-1

[B14] Uden S Bilton D Nathan L Hunt LP Main C Braganza JM Antioxidant therapy for recurrent pancreatitis: placebo-controlled trial Aliment Pharmacol Ther 1990 4 357 371 2103755 10.1111/j.1365-2036.1990.tb00482.x

[B15] Freedman SD Sakamoto K Venu RP GP2, the homologue to the renal cast protein uromodulin, is a major component of intraductal plugs in chronic pancreatitis J Clin Invest 1993 92 83 90 10.1172/JCI116602 8326020 PMC293537

[B16] Sahel J Sarles H Modifications of pure human pancreatic juice induced by chronic alcohol consumption Dig Dis Sci 1979 24 897 905 10.1007/BF01311942 510088

[B17] Kloppel G Pathology of chronic pancreatitis and pancreatic pain Acta Chir Scand 1990 156 261 265 2349844

[B18] Kloppel G Maillet B Pathology of acute and chronic pancreatitis Pancreas 1993 8 659 670 10.1097/00006676-199311000-00001 8255882

[B19] Blaine SA Ray KC Branch KM Robinson PS Whitehead RH Means AL Epidermal growth factor receptor regulates pancreatic fibrosis Am J Physiol Gastrointest Liver Physiol 2009 297 G434 441 10.1152/ajpgi.00152.2009 19608732 PMC2739824

[B20] Apte MV Haber PS Applegate TL Norton ID McCaughan GW Korsten MA Pirola RC Wilson JS Periacinar stellate shaped cells in rat pancreas: identification, isolation, and culture Gut 1998 43 128 133 10.1136/gut.43.1.128 9771417 PMC1727174

[B21] Apte M Pirola R Wilson J New insights into alcoholic pancreatitis and pancreatic cancer J Gastroenterol Hepatol 2009 24 Suppl 3 S51 56 19799699 10.1111/j.1440-1746.2009.06071.x

[B22] Gress TM Müller-Pillasch F Lerch MM Friess H Büchler M Beger HG Adler G Balance of expression of genes-coding for extracellular-matrix proteins and extracellular-matrix degrading proteases in chronic-pancreatitis Z Gastroenterol 1994 32 221 225 8017097

[B23] Mews P Phillips P Fahmy R Korsten M Pirola R Wilson J Apte M Pancreatic stellate cells respond to inflammatory cytokines: potential role in chronic pancreatitis Gut 2002 50 535 541 10.1136/gut.50.4.535 11889076 PMC1773172

[B24] Vonlaufen A Xu Z Daniel B Kumar RK Pirola R Wilson J Apte MV Bacterial endotoxin: a trigger factor for alcoholic pancreatitis? Evidence from a novel, physiologically relevant animal model Gastroenterology 2007 133 1293 1303 10.1053/j.gastro.2007.06.062 17919500

[B25] Aoki H Ohnishi H Hama K Ishijima T Satoh Y Hanatsuka K Ohashi A Wada S Miyata T Kita H Yamamoto H Osawa H Sato K Tamada K Yasuda H Mashima H Sugano K Autocrine loop between TGF-beta1 and IL-1beta through Smad3-and ERK-dependent pathways in rat pancreatic stellate cells Am J Physiol Cell Physiol 2006 290 C1100 1108 16371439 10.1152/ajpcell.00465.2005

[B26] Phillips PA Wu MJ Kumar RK Doherty E McCarroll JA Park S Pirola RC Wilson JS Apte MV Cell migration: a novel aspect of pancreatic stellate cell biology Gut 2003 52 677 682 10.1136/gut.52.5.677 12692052 PMC1773645

[B27] Shimizu K Kobayashi M Tahara J Shiratori K Cytokines and peroxisome proliferator activated receptor gamma ligand regulate phagocytosis by pancreatic stellate cells Gastroenterology 2005 128 2105 2118 10.1053/j.gastro.2005.03.025 15940641

[B28] Masamune A Kikuta K Watanabe T Satoh K Satoh A Shimosegawa T Pancreatic stellate cells express Toll-like receptors J Gastroenterol 2008 43 352 362 10.1007/s00535-008-2162-0 18592153

[B29] Völzke H Baumeister SE Alte D Hoffmann W Schwahn C Simon P John U Lerch MM Independent risk factors for gallstone formation in a region with high cholelithiasis prevalence Digestion 2005 71 97 105 10.1159/000084525 15775677

[B30] Keller J Aghdassi AA Lerch MM Mayerle JV Layer P Tests of pancreatic exocrine function-clinical significance in pancreatic and non-pancreatic disorders Best Pract Res Clin Gastroenterol 2009 23 425 439 10.1016/j.bpg.2009.02.013 19505669

[B31] Andersen DK Mechanisms and emerging treatments of the metabolic complications of chronic pancreatitis Pancreas 2007 35 1 15 10.1097/mpa.0b013e31805d01b0 17575539

[B32] Lieber CS Jones DP Decarli LM Effects of prolonged ethanol intake: production of fatty liver despite adequate diets J Clin Invest 1965 44 1009 1021 10.1172/JCI105200 14322019 PMC292581

[B33] Thrower E Husain S Gorelick F Molecular basis for pancreatitis Curr Opin Gastroenterol 2008 24 580 585 10.1097/MOG.0b013e32830b10e6 19122498 PMC3030809

[B34] Opie EL The relation of cholelithiasis to disease of the pancreas and to fat necrosis Johns Hopkins Hosp Bull 1901 12 19 21

[B35] Opie EL The etiology of acute hemorrhagic pancreatitis Johns Hopkins Hosp Bull 1901 12 182 188

[B36] Lerch MM Saluja AK Dawra R Ramarao P Saluja M Steer ML Acute necrotizing pancreatitis in the opossum: earliest morphological changes involve acinar cells Gastroenterology 1992 103 205 213 1612327 10.1016/0016-5085(92)91114-j

[B37] Lerch MM Saluja AK Runzi M Dawra R Saluja M Steer ML Pancreatic duct obstruction triggers acute necrotizing pancreatitis in the opossum Gastroenterology 1993 104 853 861 7680018 10.1016/0016-5085(93)91022-a

[B38] Seidel H Bemerkungen zu meiner Methode der experimentellen Erzeugung der akuten hämorrhagischen Pankreatitis Zentralbl der Chir 1910 2 46 59

[B39] Pfeffer RB Stasior O Hinton JW The clinical picture of the sequential development of acute hemorrhagic pancreatitis in the dog Surg Forum 1957 8 248 251 13529599

[B40] De Rai P Franciosi C Confalonieri GM Biffi R Andreoni B Uggeri F Malesci A Effects of somatostatin on acute pancreatitis induced in rats by injection of taurocholate and trypsin into a temporarily closed duodenal loop Int J Pancreatol 1988 3 367 373 2902146 10.1007/BF02788470

[B41] Klein ES Grateron H Rudick J Dreiling DA Pancreatic intraductal pressure: I. A consideration of regulatory factors Am J Gastroenterol 1983 78 507 509 6881116

[B42] Stone HH Fabian TC Dunlop WE Gallstone pancreatitis: biliary tract pathology in relation to time of operation Ann Surg 1981 194 305 312 10.1097/00000658-198109000-00008 6168240 PMC1345356

[B43] Churg A Richter WR Early changes in the exocrine pancreas of the dog and rat after ligation of the pancreatic duct. A light and electron microscopic study Am J Pathol 1971 63 521 546 5581235 PMC2047483

[B44] Watanabe S Abe K Anbo Y Katoh H Changes in the mouse exocrine pancreas after pancreatic duct ligation: a qualitative and quantitative histological study Arch Histol Cytol 1995 58 365 374 10.1679/aohc.58.365 8527243

[B45] Kimura W Histological study on pathogenesis of sites of isolated islets of Langerhans and their course to the terminal state Am J Gastroenterol 1989 84 517 522 2655435

[B46] DiMagno EP Go VL Summerskill WH Relations between pancreatic enzyme ouputs and malabsorption in severe pancreatic insufficiency N Engl J Med 1973 288 813 815 10.1056/NEJM197304192881603 4693931

[B47] Tanaka T Ichiba Y Fujii Y Itoh H Kodama O Dohi K New canine model of chronic pancreatitis due to chronic ischemia with incomplete pancreatic duct obstruction Digestion 1988 41 149 155 10.1159/000199767 3224767

[B48] Tanaka T Miura Y Matsugu Y Ichiba Y Ito H Dohi K Pancreatic duct obstruction is an aggravating factor in the canine model of chronic alcoholic pancreatitis Gastroenterology 1998 115 1248 1253 10.1016/S0016-5085(98)70097-6 9797381

[B49] Hirano T Saluja A Ramarao P Lerch MM Saluja M Steer ML Apical secretion of lysosomal-enzymes in rabbit pancreas occurs via a secretagogue regulated pathway and is increased after pancreatic duct obstruction J Clin Invest 1991 87 865 869 10.1172/JCI115091 1705567 PMC329875

[B50] Mooren FCh Hlouschek V Finkes T Turi S Weber IA Singh J Domschke W Schnekenburger J Krüger B Lerch MM Early changes in pancreatic acinar cell calcium signaling after pancreatic duct obstruction J Biol Chem 2003 278 9361 9369 10.1074/jbc.M207454200 12522141

[B51] Boerma D Straatsburg IH Offerhaus GJ Gouma DJ van Gulik TM Experimental model of obstructive, chronic pancreatitis in pigs Dig Surg 2003 20 520 526 10.1159/000073688 14534374

[B52] Kishi S Takeyama Y Ueda T Yasuda T Shinzeki M Kuroda Y Yokozaki H Pancreatic duct obstruction itself induces expression of alpha smooth muscle actin in pancreatic stellate cells J Surg Res 2003 114 6 14 10.1016/S0022-4804(03)00153-7 13678692

[B53] Isaksson G Lundquist I Ihse I Effects on the exocrine and endocrine pancreas of duct occlusion with two different tissue glues in the rat Eur Surg Res 1983 15 136 144 10.1159/000128345 6345169

[B54] Engel SRW Dockerty MB Effect of ligation of pancreatic ducts on chronic pancreatitis Arch Surg 1962 85 1031 1035 10.1001/archsurg.1962.01310060167032

[B55] Chiari H Über die Selbstverdauung des menschlichen Pankreas Z Heilk 1896 17 69 96

[B56] Kruger B Lerch MM Tessenow W Direct detection of premature protease activation in living pancreatic acinar cells Lab Invest 1998 78 763 764 9645767

[B57] Foulis AK Histological evidence of initiating factors in acute necrotising pancreatitis in man J Clin Pathol 1980 33 1125 1131 10.1136/jcp.33.12.1125 7451660 PMC1146363

[B58] Bialek R Willemer S Arnold R Adler G Evidence of intracellular activation of serine proteases in acute cerulein-induced pancreatitis in rats Scand J Gastroenterol 1991 26 190 196 10.3109/00365529109025030 1707179

[B59] Luthen R Niederau C Grendell JH Intrapancreatic zymogen activation and levels of ATP and glutathione during caerulein pancreatitis in rats Am J Physiol 1995 268 G592 604 7537455 10.1152/ajpgi.1995.268.4.G592

[B60] Rinderknecht H Activation of pancreatic zymogens. Normal activation, premature intrapancreatic activation, protective mechanisms against inappropriate activation Dig Dis Sci 1986 31 314 321 10.1007/BF01318124 2936587

[B61] Mouret J Contribution á l'étude des cellules glandulaires (pancreas) J Anat Physiol 1895 31 221 236

[B62] Adler G Gerhards G Schick J Rohr G Kern HF Effects of *in vivo *cholinergic stimulation of rat exocrine pancreas Am J Physiol 1983 244 G623 629 6190409 10.1152/ajpgi.1983.244.6.G623

[B63] Niederau C Ferrell LD Grendell JH Caerulein-induced acute necrotizing pancreatitis in mice: protective effects of proglumide, benzotript, and secretin Gastroenterology 1985 88 1192 1204 2984080 10.1016/s0016-5085(85)80079-2

[B64] Saluja A Saito I Saluja M Houlihan MJ Powers RE Meldolesi J Steer M *In vivo *rat pancreatic acinar cell function during supramaximal stimulation with caerulein Am J Physiol 1985 249 G702 710 2417493 10.1152/ajpgi.1985.249.6.G702

[B65] Watanabe O Baccino FM Steer ML Meldolesi J Supramaximal caerulein stimulation and ultrastructure of rat pancreatic acinar cell: early morphological changes during development of experimental pancreatitis Am J Physiol 1984 246 G457 467 6720895 10.1152/ajpgi.1984.246.4.G457

[B66] Pantoja JL Renner IG Abramson SB Edmondson HA Production of acute hemorrhagic pancreatitis in the dog using venom of the scorpion, Buthus quinquestriatus Dig Dis Sci 1983 28 429 439 10.1007/BF02430532 6839906

[B67] Ji B Bi Y Simeone D Mortensen RM Logsdon CD Human pancreatic acinar cells do not respond to cholecystokinin Pharmacol Toxicol 2002 91 327 332 10.1034/j.1600-0773.2002.910610.x 12688376

[B68] Lampel M Kern HF Acute interstitial pancreatitis in the rat induced by excessive doses of a pancreatic secretagogue Virchows Arch A Pathol Anat Histol 1977 373 97 117 10.1007/BF00432156 139754

[B69] Scheele GA Palade GE Studies on the guinea pig pancreas. Parallel discharge of exocrine enzyme activities J Biol Chem 1975 250 2660 2670 1123325

[B70] Lerch MM Albrecht E Ruthenburger M Mayerle J Halangk W Kruger B Pathophysiology of alcohol-induced pancreatitis Pancreas 2003 27 291 296 10.1097/00006676-200311000-00003 14576489

[B71] Lerch MM Lutz MP Weidenbach H Muller-Pillasch F Gress TM Leser J Adler G Dissociation and reassembly of adherens junctions during experimental acute pancreatitis Gastroenterology 1997 113 1355 1366 10.1053/gast.1997.v113.pm9322531 9322531

[B72] Lerch MM Saluja AK Runzi M Dawra R Steer ML Luminal endocytosis and intracellular targeting by acinar cells during early biliary pancreatitis in the opossum J Clin Invest 1995 95 2222 2231 10.1172/JCI117912 7537759 PMC295834

[B73] Lerch MM Saluja AK Dawra R Saluja M Steer ML The effect of chloroquine administration on two experimental models of acute pancreatitis Gastroenterology 1993 104 1768 1779 8500736 10.1016/0016-5085(93)90658-y

[B74] Adler G Rohr G Kern HF Alteration of membrane fusion as a cause of acute pancreatitis in the rat Dig Dis Sci 1982 27 993 1002 10.1007/BF01391745 7140496

[B75] Shimizu K Mechanisms of pancreatic fibrosis and applications to the treatment of chronic pancreatitis J Gastroenterol 2008 43 823 832 10.1007/s00535-008-2249-7 19012035

[B76] Gress T Muller-Pillasch F Elsasser HP Bachem M Ferrara C Weidenbach H Lerch M Adler G Enhancement of transforming growth factor beta 1 expression in the rat pancreas during regeneration from caerulein-induced pancreatitis Eur J Clin Invest 1994 24 679 685 10.1111/j.1365-2362.1994.tb01060.x 7851468

[B77] Menke A Yamaguchi H Gress TM Adler G Extracellular matrix is reduced by inhibition of transforming growth factor beta1 in pancreatitis in the rat Gastroenterology 1997 113 295 303 10.1016/S0016-5085(97)70107-0 9207290

[B78] Neuschwander-Tetri BA Burton FR Presti ME Britton RS Janney CG Garvin PR Brunt EM Galvin NJ Poulos JE Repetitive self-limited acute pancreatitis induces pancreatic fibrogenesis in the mouse Dig Dis Sci 2000 45 665 674 10.1023/A:1005423122127 10759232

[B79] Iovanna JL Odaira C Berger Z Sarles H Temporary pseudochronic lesions during the recovery of acute necrohemorrhagic pancreatitis in rabbits Pancreas 1988 3 433 438 10.1097/00006676-198808000-00011 3174606

[B80] Bockman DE Boydston WR Anderson MC Origin of tubular complexes in human chronic pancreatitis Am J Surg 1982 144 243 249 10.1016/0002-9610(82)90518-9 7102934

[B81] Miyahara T Kawabuchi M Goto M Nakano I Nada O Nawata H Morphological study of pancreatic endocrine in an experimental chronic pancreatitis with diabetes induced by stress and cerulein Ultrastruct Pathol 1999 23 171 180 10.1080/019131299281671 10445284

[B82] Goto M Nakano I Kimura T Miyahara T Kinjo M Nawata H New chronic pancreatitis model with diabetes induced by cerulein plus stress in rats Dig Dis Sci 1995 40 2356 2363 10.1007/BF02063237 7587814

[B83] Ohashi S Nishio A Nakamura H Asada M Tamaki H Kawasaki K Fukui T Yodoi J Chiba T Overexpression of redox-active protein thioredoxin-1 prevents development of chronic pancreatitis in mice Antioxid Redox Signal 2006 8 1835 1845 10.1089/ars.2006.8.1835 16987036

[B84] Sugiyama M Kobori O Atomi Y Wada N Kuroda A Muto T Pancreatic exocrine function during acute exacerbation in WBN/Kob rats with spontaneous chronic pancreatitis Int J Pancreatol 1996 20 191 196 10.1007/BF02803768 9013280

[B85] Elsasser HP Haake T Grimmig M Adler G Kern HF Repetitive cerulein-induced pancreatitis and pancreatic fibrosis in the rat Pancreas 1992 7 385 390 10.1097/00006676-199205000-00017 1594561

[B86] Miyamoto T Nakamura H Nagashio Y Asaumi H Harada M Otsuki M Overexpression of Smad6 exacerbates pancreatic fibrosis in murine caerulein-induced chronic pancreatic injuries Pancreas 2010 39 385 391 10.1097/MPA.0b013e3181bb9603 19823096

[B87] Dawra R Sharif R Phillips P Dudeja V Dhaulakhandi D Saluja AK Development of a new mouse model of acute pancreatitis induced by administration of l-arginine Am J Physiol Gastrointest Liver Physiol 2007 292 G1009 1018 17170029 10.1152/ajpgi.00167.2006

[B88] Takacs T Czako L Morschl E Laszlo F Tiszlavicz L Rakonczay Z Jr Lonovics J The role of nitric oxide in edema formation in l-arginine-induced acute pancreatitis Pancreas 2002 25 277 282 10.1097/00006676-200210000-00010 12370539

[B89] Hegyi P Rakonczay Z Jr Sari R Gog C Lonovics J Takacs T Czako L l-Arginine induced experimental pancreatitis World J Gastroenterol 2004 10 2003 2009 15237423 10.3748/wjg.v10.i14.2003 PMC4572322

[B90] Mizunuma T Kawamura S Kishino Y Effects of injecting excess arginine on rat pancreas J Nutr 1984 114 467 471 6199486 10.1093/jn/114.3.467

[B91] Tani S Itoh H Okabayashi Y Nakamura T Fujii M Fujisawa T Koide M Otsuki M New model of acute necrotizing pancreatitis induced by excessive doses of arginine in rats Dig Dis Sci 1990 35 367 374 10.1007/BF01537416 2307082

[B92] Delaney CP McGeeney KF Dervan P Fitzpatrick JM Pancreatic atrophy: a new model using serial intra-peritoneal injections of l-arginine Scand J Gastroenterol 1993 28 1086 1090 10.3109/00365529309098314 8303212

[B93] Weaver C Bishop AE Polak JM Pancreatic changes elicited by chronic administration of excess l-arginine Exp Mol Pathol 1994 60 71 87 10.1006/exmp.1994.1007 8070543

[B94] Yamaguchi T Kihara Y Taguchi M Nagashio Y Tashiro M Nakamura H Otsuki M Persistent destruction of the basement membrane of the pancreatic duct contributes to progressive acinar atrophy in rats with experimentally induced pancreatitis Pancreas 2005 31 365 372 10.1097/01.mpa.0000179729.61457.e5 16258372

[B95] Wright SD Ramos RA Tobias PS Ulevitch RJ Mathison JC CD14, a receptor for complexes of lipopolysaccharide (LPS) and LPS binding protein Science 1990 249 1431 1433 10.1126/science.1698311 1698311

[B96] Poltorak A He X Smirnova I Liu MY Van Huffel C Du X Birdwell D Alejos E Silva M Galanos C Freudenberg M Ricciardi-Castagnoli P Layton B Beutler B Defective LPS signaling in C3H/HeJ and C57BL/10ScCr mice: mutations in Tlr4 gene Science 1998 282 2085 2088 9851930 10.1126/science.282.5396.2085

[B97] Nagai Y Akashi S Nagafuku M Ogata M Iwakura Y Akira S Kitamura T Kosugi A Kimoto M Miyake K Essential role of MD-2 in LPS responsiveness and TLR4 distribution Nat Immunol 2002 3 667 672 12055629 10.1038/ni809

[B98] Takeuchi O Takeda K Hoshino K Adachi O Ogawa T Akira S Cellular responses to bacterial cell wall components are mediated through MyD88-dependent signaling cascades Int Immunol 2000 12 113 117 10.1093/intimm/12.1.113 10607756

[B99] Kawai T Adachi O Ogawa T Takeda K Akira S Unresponsiveness of MyD88-deficient mice to endotoxin Immunity 1999 11 115 122 10.1016/S1074-7613(00)80086-2 10435584

[B100] Zandi E Rothwarf DM Delhase M Hayakawa M Karin M The IkappaB kinase complex (IKK) contains two kinase subunits, IKKalpha and IKKbeta, necessary for IkappaB phosphorylation and NF-kappaB activation Cell 1997 91 243 252 10.1016/S0092-8674(00)80406-7 9346241

[B101] Ding SP Li JC Jin C A mouse model of severe acute pancreatitis induced with caerulein and lipopolysaccharide World J Gastroenterol 2003 9 584 589 12632523 10.3748/wjg.v9.i3.584 PMC4621587

[B102] Segersvard R Sylvan M Lempinen M Larsson J Permert J Impact of chronic and acute high-fat feeding on acute experimental pancreatitis complicated by endotoxinaemia Scand J Gastroenterol 2004 39 74 80 10.1080/00365520310007233 14992565

[B103] Vaquero E Molero X Tian X Salas A Malagelada JR Myofibroblast proliferation, fibrosis, and defective pancreatic repair induced by cyclosporin in rats Gut 1999 45 269 277 10.1136/gut.45.2.269 10403741 PMC1727609

[B104] Gukovsky I Lugea A Shahsahebi M Cheng JH Hong PP Jung YJ Deng QG French BA Lungo W French SW Tsukamoto H Pandol SJ A rat model reproducing key pathological responses of alcoholic chronic pancreatitis Am J Physiol Gastrointest Liver Physiol 2008 294 G68 79 17884979 10.1152/ajpgi.00006.2007

[B105] Hirakawa K Ohkuma S Kuriyama K Functional and morphological changes of the exocrine pancreas in ciclosporin-treated rats Eur Surg Res 1991 23 292 301 10.1159/000129167 1724964

[B106] Sparmann G Merkord J Jaschke A Nizze H Jonas L Lohr M Liebe S Emmrich J Pancreatic fibrosis in experimental pancreatitis induced by dibutyltin dichloride Gastroenterology 1997 112 1664 1672 10.1016/S0016-5085(97)70049-0 9136846

[B107] Merkord J Weber H Kroning G Hennighausen G Repeated administration of a mild acute toxic dose of di-*N*-butyltin dichloride at intervals of 3 weeks induces severe lesions in pancreas and liver of rats Hum Exp Toxicol 2001 20 386 392 10.1191/096032701682692964 11727788

[B108] Merkord J Weber H Sparmann G Jonas L Hennighausen G The course of pancreatic fibrosis induced by dibutyltin dichloride (DBTC) Ann N Y Acad Sci 1999 880 231 237 10.1111/j.1749-6632.1999.tb09527.x 10415868

[B109] Leung PS Chan YC Role of oxidative stress in pancreatic inflammation Antioxid Redox Signal 2009 11 135 165 10.1089/ars.2008.2109 18837654

[B110] Wildi S Kleeff J Mayerle J Zimmermann A Böttinger EP Wakefield L Büchler MW Friess H Korc M Suppression of transforming growth factor beta signalling aborts caerulein induced pancreatitis and eliminates restricted stimulation at high caerulein concentrations Gut 2007 56 685 692 10.1136/gut.2006.105833 17135311 PMC1942167

[B111] He J Sun X Qian KQ Liu X Wang Z Chen Y Protection of cerulein-induced pancreatic fibrosis by pancreas-specific expression of Smad7 Biochim Biophys Acta 2009 1792 56 60 19015026 10.1016/j.bbadis.2008.10.010

[B112] Li J Guo M Hu B Liu R Wang R Tang C Does chronic ethanol intake cause chronic pancreatitis?: evidence and mechanism Pancreas 2008 37 189 195 10.1097/MPA.0b013e31816459b7 18665082

[B113] Vonlaufen A Wilson JS Apte MV Molecular mechanisms of pancreatitis: current opinion J Gastroenterol Hepatol 2008 23 1339 1348 10.1111/j.1440-1746.2008.05520.x 18853993

[B114] Gukovskaya AS Mouria M Gukovsky I Reyes CN Kasho VN Faller LD Pandol SJ Ethanol metabolism and transcription factor activation in pancreatic acinar cells in rats Gastroenterology 2002 122 106 118 10.1053/gast.2002.30302 11781286

[B115] Ponnappa BC Marciniak R Schneider T Hoek JB Rubin E Ethanol consumption and susceptibility of the pancreas to cerulein-induced pancreatitis Pancreas 1997 14 150 157 10.1097/00006676-199703000-00007 9057187

[B116] Lieber CS DeCarli LM Alcoholic liver injury: experimental models in rats and baboons Adv Exp Med Biol 1975 59 379 393 1237225 10.1007/978-1-4757-0632-1_27

[B117] Lieber CS Decarli LM Animal models of ethanol dependence and liver injury in rats and baboons Fed Proc 1976 35 1232 1236 944146

[B118] Sarles H Lebreuil G Tasso F Figarella C Clemente F Devaux MA Fagonde B Payan H A comparison of alcoholic pancreatitis in rat and man Gut 1971 12 377 388 10.1136/gut.12.5.377 4329553 PMC1411611

[B119] Tsukamoto H Towner SJ Yu GS French SW Potentiation of ethanol-induced pancreatic injury by dietary fat. Induction of chronic pancreatitis by alcohol in rats Am J Pathol 1988 131 246 257 3358454 PMC1880607

[B120] Deng X Wang L Elm MS Gabazadeh D Diorio GJ Eagon PK Whitcomb DC Chronic alcohol consumption accelerates fibrosis in response to cerulein-induced pancreatitis in rats Am J Pathol 2005 166 93 106 10.1016/S0002-9440(10)62235-3 15632003 PMC1602301

[B121] Perides G Tao X West N Sharma A Steer ML A mouse model of ethanol dependent pancreatic fibrosis Gut 2005 54 1461 1467 10.1136/gut.2004.062919 15870229 PMC1774704

[B122] Perkins PS Rutherford RE Pandol SJ Effect of chronic ethanol feeding on digestive enzyme synthesis and mRNA content in rat pancreas Pancreas 1995 10 14 21 10.1097/00006676-199501000-00002 7899455

[B123] Kobori O Gedigk P Totovic V Adenomatous changes and adenocarcinoma of glandular stomach in Wistar rats induced by *N*-methyl-*N*'-nitro-*N*-nitrosoguanidine. An electron microscopic and histochemical study Virchows Arch A Pathol Anat Histol 1977 373 37 54 10.1007/BF00432467 139023

[B124] Tsuchitani M Saegusa T Narama I Nishikawa T Gonda T A new diabetic strain of rat (WBN/Kob) Lab Anim 1985 19 200 207 10.1258/002367785780893575 4033061

[B125] Ohashi K Kim JH Hara H Aso R Akimoto T Nakama K WBN/Kob rats. A new spontaneously occurring model of chronic pancreatitis Int J Pancreatol 1990 6 231 247 1698893

[B126] Hashimoto T Yamada T Yokoi T Sano H Ando H Nakazawa T Ohara H Nomura T Joh T Itoh M Apoptosis of acinar cells is involved in chronic pancreatitis in Wbn/Kob rats: role of glucocorticoids Pancreas 2000 21 296 304 10.1097/00006676-200010000-00012 11039475

[B127] Mori M Fu X Chen L Zhang G Higuchi K Hereditary pancreatitis model WBN/Kob rat strain has a unique haplotype in the Pdwk1 region on chromosome 7 Exp Anim 2009 58 409 413 10.1538/expanim.58.409 19654439

[B128] O'Toole JF Liu Y Davis EE Westlake CJ Attanasio M Otto EA Seelow D Nurnberg G Becker C Nuutinen M Kärppä M Ignatius J Uusimaa J Pakanen S Jaakkola E van den Heuvel LP Fehrenbach H Wiggins R Goyal M Zhou W Wolf MT Wise E Helou J Allen SJ Murga-Zamalloa CA Ashraf S Chaki M Heeringa S Chernin G Hoskins BE Individuals with mutations in XPNPEP3, which encodes a mitochondrial protein, develop a nephronophthisis-like nephropathy J Clin Invest 2010 120 791 802 10.1172/JCI40076 20179356 PMC2827951

[B129] Whitcomb DC Gorry MC Preston RA Furey W Sossenheimer MJ Ulrich CD Martin SP Gates LK Jr Amann ST Toskes PP Liddle R McGrath K Uomo G Post JC Ehrlich GD Hereditary pancreatitis is caused by a mutation in the cationic trypsinogen gene Nat Genet 1996 14 141 145 10.1038/ng1096-141 8841182

[B130] Selig L Sack U Gaiser S Kloppel G Savkovic V Mossner J Keim V Bodeker H Characterisation of a transgenic mouse expressing R122H human cationic trypsinogen BMC Gastroenterol 2006 6 30 10.1186/1471-230X-6-30 17069643 PMC1637108

[B131] Archer H Jura N Keller J Jacobson M Bar-Sagi D A mouse model of hereditary pancreatitis generated by transgenic expression of R122H trypsinogen Gastroenterology 2006 131 1844 1855 10.1053/j.gastro.2006.09.049 17087933

[B132] Gaiser S Daniluk J Liu Y Tsou L Chu J Lee W Longnecker DS Logsdon CD Ji B Intracellular activation of trypsinogen in transgenic mice induces acute but not chronic pancreatitis Gut 2011 60 1379 1388 10.1136/gut.2010.226175 21471572 PMC4304390

[B133] Ellis I Lerch MM Whitcomb DC Genetic testing for hereditary pancreatitis: Guidelines for indications, counselling, consent and privacy issues Pancreatology 2001 1 405 415 10.1159/000055840 12120217

[B134] Keim V Bauer N Teich N Simon P Lerch MM Mössner J Clinical characterization of patients with hereditary pancreatitis and mutations in the cationic trypsinogen gene Am J Med 2001 111 622 626 10.1016/S0002-9343(01)00958-5 11755505

[B135] Ohmuraya M Hirota M Araki K Baba H Yamamura K Enhanced trypsin activity in pancreatic acinar cells deficient for serine protease inhibitor kazal type 3 Pancreas 2006 33 104 106 10.1097/01.mpa.0000226889.86322.9b 16804421

[B136] Ohmuraya M Hirota M Araki M Mizushima N Matsui M Mizumoto T Haruna K Kume S Takeya M Ogawa M Araki K Yamamura K Autophagic cell death of pancreatic acinar cells in serine protease inhibitor Kazal type 3-deficient mice Gastroenterology 2005 129 696 705 16083722 10.1016/j.gastro.2005.05.057

[B137] Nathan JD Romac J Peng RY Peyton M Macdonald RJ Liddle RA Transgenic expression of pancreatic secretory trypsin inhibitor-I ameliorates secretagogue-induced pancreatitis in mice Gastroenterology 2005 128 717 727 10.1053/j.gastro.2004.11.052 15765407

[B138] Riordan JR Rommens JM Kerem B Alon N Rozmahel R Grzelczak Z Zielenski J Lok S Plavsic N Chou JL Drumm ML Iannuzzi MC Collins FS Tsui LC Identification of the cystic fibrosis gene: cloning and characterization of complementary DNA Science 1989 245 1066 1073 10.1126/science.2475911 2475911

[B139] Durno C Corey M Zielenski J Tullis E Tsui LC Durie P Genotype and phenotype correlations in patients with cystic fibrosis and pancreatitis Gastroenterology 2002 123 1857 1864 10.1053/gast.2002.37042 12454843

[B140] Snouwaert JN Brigman KK Latour AM Malouf NN Boucher RC Smithies O Koller BH An animal model for cystic fibrosis made by gene targeting Science 1992 257 1083 1088 10.1126/science.257.5073.1083 1380723

[B141] De Lisle RC Isom KS Ziemer D Cotton CU Changes in the exocrine pancreas secondary to altered small intestinal function in the CF mouse Am J Physiol Gastrointest Liver Physiol 2001 281 G899 906 11557509 10.1152/ajpgi.2001.281.4.G899

[B142] Dimagno MJ Lee SH Hao Y Zhou SY McKenna BJ Owyang C A proinflammatory, antiapoptotic phenotype underlies the susceptibility to acute pancreatitis in cystic fibrosis transmembrane regulator (-/-) mice Gastroenterology 2005 129 665 681 16083720 10.1016/j.gastro.2005.05.059

[B143] Rogers CS Stoltz DA Meyerholz DK Ostedgaard LS Rokhlina T Taft PJ Rogan MP Pezzulo AA Karp PH Itani OA Kabel AC Wohlford-Lenane CL Davis GJ Hanfland RA Smith TL Samuel M Wax D Murphy CN Rieke A Whitworth K Uc A Starner TD Brogden KA Shilyansky J McCray PB Jr Zabner J Prather RS Welsh MJ Disruption of the CFTR gene produces a model of cystic fibrosis in newborn pigs Science 2008 321 1837 1841 10.1126/science.1163600 18818360 PMC2570747

[B144] Cano DA Sekine S Hebrok M Primary cilia deletion in pancreatic epithelial cells results in cyst formation and pancreatitis Gastroenterology 2006 131 1856 1869 10.1053/j.gastro.2006.10.050 17123526

[B145] Lin F Hiesberger T Cordes K Sinclair AM Goldstein LS Somlo S Igarashi P Kidney specific inactivation of the KIF3A subunit of kinesin-II inhibits renal ciliogenesis and produces polycystic kidney disease Proc Natl Acad Sci USA 2003 100 5286 5291 10.1073/pnas.0836980100 12672950 PMC154337

[B146] Harding HP Zeng H Zhang Y Jungries R Chung P Plesken H Sabatini DD Ron D Diabetes mellitus and exocrine pancreatic dysfunction in perk-/-mice reveals a role for translational control in secretory cell survival Mol Cell 2001 7 1153 1163 10.1016/S1097-2765(01)00264-7 11430819

[B147] Marrache F Tu SP Bhagat G Pendyala S Osterreicher CH Gordon S Ramanathan V Penz-Osterreicher M Betz KS Song Z Wang TC Overexpression of interleukin-1beta in the murine pancreas results in chronic pancreatitis Gastroenterology 2008 135 1277 1287 10.1053/j.gastro.2008.06.078 18789941 PMC2707078

[B148] Norman JG Fink GW Sexton C Carter G Transgenic animals demonstrate a role for the IL-1 receptor in regulating IL-1beta gene expression at steady-state and during the systemic stress induced by acute pancreatitis J Surg Res 1996 63 231 236 10.1006/jsre.1996.0253 8661203

[B149] Hunger RE Mueller C Z'Graggen K Friess H Buchler MW Cytotoxic cells are activated in cellular infiltrates of alcoholic chronic pancreatitis Gastroenterology 1997 112 1656 1663 10.1016/S0016-5085(97)70048-9 9136845

